# Association of aging-related genes with prognosis and immune infiltration in pancreatic adenocarcinoma

**DOI:** 10.3389/fcell.2022.942225

**Published:** 2022-08-08

**Authors:** Shengbai Xue, Weiyu Ge, Kexuan Wang, Tiebo Mao, Xiaofei Zhang, Haiyan Xu, Yongchao Wang, Jiayu Yao, Shumin Li, Ming Yue, Jingyu Ma, Yanling Wang, Daiyuan Shentu, Jiujie Cui, Liwei Wang

**Affiliations:** ^1^ Department of Oncology, Renji Hospital, School of Medicine, Shanghai Jiaotong University, Shanghai, China; ^2^ Department of Nursing, School of Nursing, Xuzhou Medical University, Xuzhou, China

**Keywords:** pancreatic adenocarcinoma, aging, molecular subtype, prognostic signature, immune microenviroment

## Abstract

Pancreatic adenocarcinoma (PAAD) is one of the deadliest malignancies. Aging is described as the degeneration of physiological function, which is complexly correlated with cancer. It is significant to explore the influences of aging-related genes (ARGs) on PAAD. Based on The Cancer Genome Atlas (TCGA) and Genotype-Tissue Expression (GTEx) datasets, we used univariate Cox regression analysis and acquired eight differentially expressed ARGs with prognostic values. Two molecular subtypes were identified based on these ARGs to depict PAAD patients’ overall survival (OS) and immune microenvironments preliminarily. Cluster 1 had a poor OS as well as a worse immune microenvironment. Through least absolute shrinkage and selection operator (LASSO) regression analysis, we constructed a seven-ARG risk signature based on the TCGA dataset and verified it in Gene Expression Omnibus (GEO) and International Cancer Genome Consortium (ICGC) to predict the prognoses, immune microenvironments, signal pathways, tumor mutations, and drug sensitivity of PAAD patients. The high-risk group possessed an unfavorable OS compared with that of the low-risk group. We also verified the independence and clinical availability of the risk signature by Cox regression analyses and the establishment of a nomogram, respectively. The higher risk score was associated with several clinical factors such as higher grade and advanced tumor stage as well as lower immunoscore and cluster 1. The negative associations of risk scores with immune, stroma, and estimate scores proved the terrible immune microenvironment in the high-risk group. Relationships between risk score and immune checkpoint gene expression as well as signal pathways provided several therapeutic targets. PAAD patients in the low-risk group possessed lower tumor mutations as well as a higher susceptibility to axitinib and vorinostat. The high-risk group bore a higher TMB and cisplatin and dasatinib may be better options. We used immunohistochemistry and qPCR to confirm the expression of key ARGs with their influences on OS. In conclusion, we identified two ARG-mediated molecular subtypes and a novel seven-ARG risk signature to predict prognoses, immune microenvironments, signal pathways, tumor mutations, and drug sensitivity of PAAD patients.

## Introduction

As a lethal malignant tumor, pancreatic adenocarcinoma (PAAD) remains the fourth-leading cause of cancer-related death globally with overall survival (OS) of less than 10% (Mizrahi et al., 2020). Surgery is considered the only potential way to cure PAAD (Okasha et al., 2017). However, late detection results in most patients being failed to tolerate surgery. Recently, immunotherapy has become a novel treatment option. Including immune checkpoint inhibitors (ICIs), tumor vaccines, and monoclonal antibodies, immunotherapy has shown unique efficacy in PAAD (Schizas et al., 2020). However, PAAD was described as a “cold tumor” with a terrible immune microenvironment (Clark et al., 2007). Therefore, it is necessary to explore novel biomarkers for predicting survival outcomes as well as immune microenvironment infiltrations of PAAD patients.

Aging, considered a gradual break of physiological integrity, has been a crucial risk factor for cancer development (López-Otín et al., 2013). Older people take up a large proportion of cancer patients, and cancer has been the first cause of death in people aged 60–79 years (Siegel et al., 2022). The interaction between aging and cancer has been studied widely. Accumulation of DNA damage, oxidative stress, and senescence-associated secretory phenotype secretion associated with aging contribute to initial tumorigenesis (Berben et al., 2021). The decline of immune functions caused by aging also leads to the increasing incidence of cancer (Camous et al., 2012). In PAAD, a previous study has found the aging of normal fibroblasts promoted the progression of cancer (Sarsour et al., 2020). However, the relationships between aging-related genes (ARGs) and prognoses as well as immune infiltrations of PAAD patients remain unclear. In previous studies, ferroptosis-related signatures (Jiang et al., 2021), immune-related signatures (Wang et al., 2020), and glycolysis-related signatures (Song et al., 2021) have been constructed to predict the OS of PAAD patients. Therefore, in this study, our purpose was to identify molecular subtypes as well as establish a risk signature based on ARGs to predict prognoses and immune microenvironments of PAAD patients.

## Materials and methods

### Public datasets

RNA sequencing data and relevant clinical features of PAAD patients (Table 1) were obtained from The Cancer Genome Atlas (TCGA), Genotype-Tissue Expression project (GTEx), Gene Expression Omnibus (GEO), and International Cancer Genome Consortium (ICGC). The expression data from TCGA, GEO, and ICGC was processed by Perl software and normalized by the R package “sva.” Patients with incomplete clinical characteristics or unavailable expression profiles were excluded. The list of ARGs (Supplementary Table S1) was achieved from the Human Ageing Genomic Resources (HAGR). Immunohistochemical results were obtained from Human Protein Atlas (HPA).

**TABLE 1:** Clinical characteristics of pancreatic adenocarcinoma patients from TCGA, GEO, and ICGC.

Clinical variables	TCGA		GSE57495		ICGC-PACA-AU	
	Number	%	Number	%	Number	%
Total	177	100	63	100	80	100
Age						
≤65y	93	52.54	—	—	—	—
>65y	84	47.46	—	—	—	—
Gender						
Male	97	54.80	—	—	—	—
Female	80	45.20	—	—	—	—
Grade						
1	31	17.51	—	—	—	—
2	94	53.11	—	—	—	—
3	48	27.12	—	—	—	—
4	2	1.13	—	—	—	—
X	2	1.13	—	—	—	—
Stage						
I	21	11.87	—	—	—	—
II	146	82.49	—	—	—	—
III	3	1.69	—	—	—	—
IV	4	2.26	—	—	—	—
Unknown	3	1.69	—	—	—	—
Tumor classification						
T1	7	3.96	—	—	—	—
T2	24	13.56	—	—	—	—
T3	141	79.66	—	—	—	—
T4	3	1.69	—	—	—	—
Tx	2	1.13	—	—	—	—
Node classification						
N0	49	27.68	—	—	—	—
N1	123	69.49	—	—	—	—
N2	0	0	—	—	—	—
N3	0	0	—	—	—	—
Nx	5	2.82	—	—	—	—
Metastasis classification						
M0	79	44.63	—	—	—	—
M1	4	2.26	—	—	—	—
Mx	94	53.11	—	—	—	—
Survival time						
≤=3y	161	90.96	54	85.71	75	93.75
>3y	16	9.04	9	14.29	5	6.25
Survival status						
Survival	89	50.28	21	33.33	32	40.00
Death	88	49.72	42	66.67	48	60.00

### Identification of differentially expressed aging-related genes with prognostic ability

By R package “limma,” we merged and normalized the data from TCGA and GTEx by averaging the multiple expressions of the same genes. We also used it to perform differential analysis between tumor samples from the TCGA dataset (*n* = 178) and normal samples from TCGA (*n* = 4) and GTEx (*n* = 167) datasets with the criteria of |log2FC| ≥ 2 as well as false discovery rate (FDR) <0.05. Taking the intersection of ARGs with differentially expressed genes (DEGs), selected differentially expressed ARGs were involved in univariate Cox regression analysis for the identification of prognostic ARGs (*p* < 0.05).

### Analyses of molecular subtypes

To identify the potential functions of prognostic ARGs, 177 patients from the TCGA dataset were classified into two clusters using the “ConsensusClusterPlus” R package (Seiler et al., 2010). The survival conditions of two subtypes were compared by the R package “survival.” Furthermore, we estimated the immune infiltration of each molecular subtype. The main categories of immune cells in PC were obtained using “CIBERSORT R script v1.03.” A comparison of expression of programmed cell death-ligand 1 (PD-L1) was conducted between two clusters. We also investigated the correlation of prognostic ARGs with *PD-L1*. Finally, we explored the expressions of the eight ARGs and clinical features in two molecular subtypes.

### Construction and validation of an aging-related gene risk signature

PAAD patients from TCGA cohort (*n* = 177) were involved in training set, while GSE57495 (*n* = 63) and ICGC-PACA-AU (*n* = 80) were selected as validation sets. RNA expression data from different datasets were normalized by the R package “sva.” To develop and validate an ARG risk signature, the least absolute shrinkage and selection operator (LASSO) regression analysis was adopted to explore key ARGs most tightly associated with OS. The risk score was computed by the formula:

risk score=∑coefficient × ARG expression

According to the median risk score, PAAD patients from the training set were separated into low-risk and high-risk subgroups. Furthermore, we performed the survival analysis by R package “survival” to find whether there existed a significant difference between the two groups. To confirm the stability of ARG risk signature at 1-, 2- and 3-year, receiver operating characteristic (ROC) curves were implemented. Afterward, analyses in validation sets validated our aforementioned results.

### Association of risk score with molecular subtypes, immunoscore, and clinicopathological characteristics

Univariate and multivariate Cox regression analyses were taken to verify the independent prognostic value of our risk signature. Prognostic factors with *p* < 0.05 in both analyses were considered independent. For clinical use to predict outcomes of PAAD patients, a nomogram with a risk score and other clinicopathological signatures was established. We drew 1-, 2-, and 3- year calibration curves to confirm its prognostic precision. Moreover, we explored the relationships between risk score and molecular subtypes, ImmuneScore as well as clinical characteristics based on the TCGA cohort. ImmuneScore of each patient was acquired by the R package “estimate.”

### Identification of immune microenvironment affected by aging-related genes

Several acknowledged methods were used to explore the relevance between risk score and immune microenvironment, including XCELL, TIMER, QUANTISEQ, MCPOUNTER, EPIC, CIBERSORT-ABS, and CIBERSORT. In addition, we evaluated the correlations of risk score and prognostic ARGs with immune checkpoint genes by the R package “limma.”

### Correlation of risk signature with signal pathways, tumor mutation, and chemosensitivity

For investigation of the relevance between signal pathways and risk score, we performed Gene Set Variation Analysis (GSVA), and the result was visualized by R packages “reshape2” and “ggplot”. Moreover, we used GeneMANIA (http://genemania.org/) to identify functionally similar genes of key ARGs and their relationships. Mutations and genetic alterations of key ARGs were visualized by cBioPortal. With the help of the R package “maftools,” we compared the mutations of the top 20 genes between two risk groups. Tumor mutation burden (TMB) scores of PAAD patients were computed by somatic mutation analysis. We also evaluated the correlation of risk score with TMB. Finally, we analyzed the survival outcomes of patients from two risk groups with different TMBs. To assess the ability of risk signature to predict chemosensitivity of PAAD, we investigated therapeutic half inhibitory centration (IC50) in two risk groups using the R package “pRRophetic”.

### Expression level validation and survival analysis of aging-related genes expression

Immunohistochemical results of ARGs involved in the risk signature were obtained from HPA for validation of ARGs expression in normal and tumor tissue. We furtherly investigated their influences on survival outcomes of PAAD patients based on the TCGA dataset. By RT-qPCR (Real Time Quantitative Polymerase Chain Reaction), we verified the expressions of two key ARGs (*BSCL2*: HR = 0.653, Coef = −0.13, and *TOP2A*: HR = 1.104, Coef = 0.07) in normal pancreatic cell line HPNE and two pancreatic adenocarcinoma cell lines PANC1 and CFPAC1. RT-qPCR was performed by a LightCycler^®^480 real-time PCR system. The ARGs’ primer sequences were shown below: *BSCL2*-forward 5′-3′ GTC​TGT​CTT​CCT​CTA​TGG​CTC​C, *BSCL2*-reverse 5′-3′ CCT​TAG​TCA​GCG​AGA​CAT​TGG​C; *TOP2A*-forward 5′-3′ ACC​ATT​GCA​GCC​TGT​AAA​TGA and *TOP2A*-reverse 5′-3′ GGG​CGG​AGC​AAA​ATA​TGT​TCC. The expression level of each gene was calculated using the 2^−ΔCT^ method.

### Statistical analysis

R software (4.1.1) and relevant packages were used for data normalization and statistical analyses. The significant standard was listed as *p* < 0.05. Independent prognostic factor was identified using Cox regression analyses. ROC curves determined the precision of our risk signature. The difference in OS was analyzed by log-rank tests and visualized by Kaplan-Meier curves. Our research was completed in accordance with the Declaration of Helsinki as revised in 2013.

## Results

### Identification of prognostic aging-related genes expressing differentially in pancreatic adenocarcinoma

**FIGURE 1:**
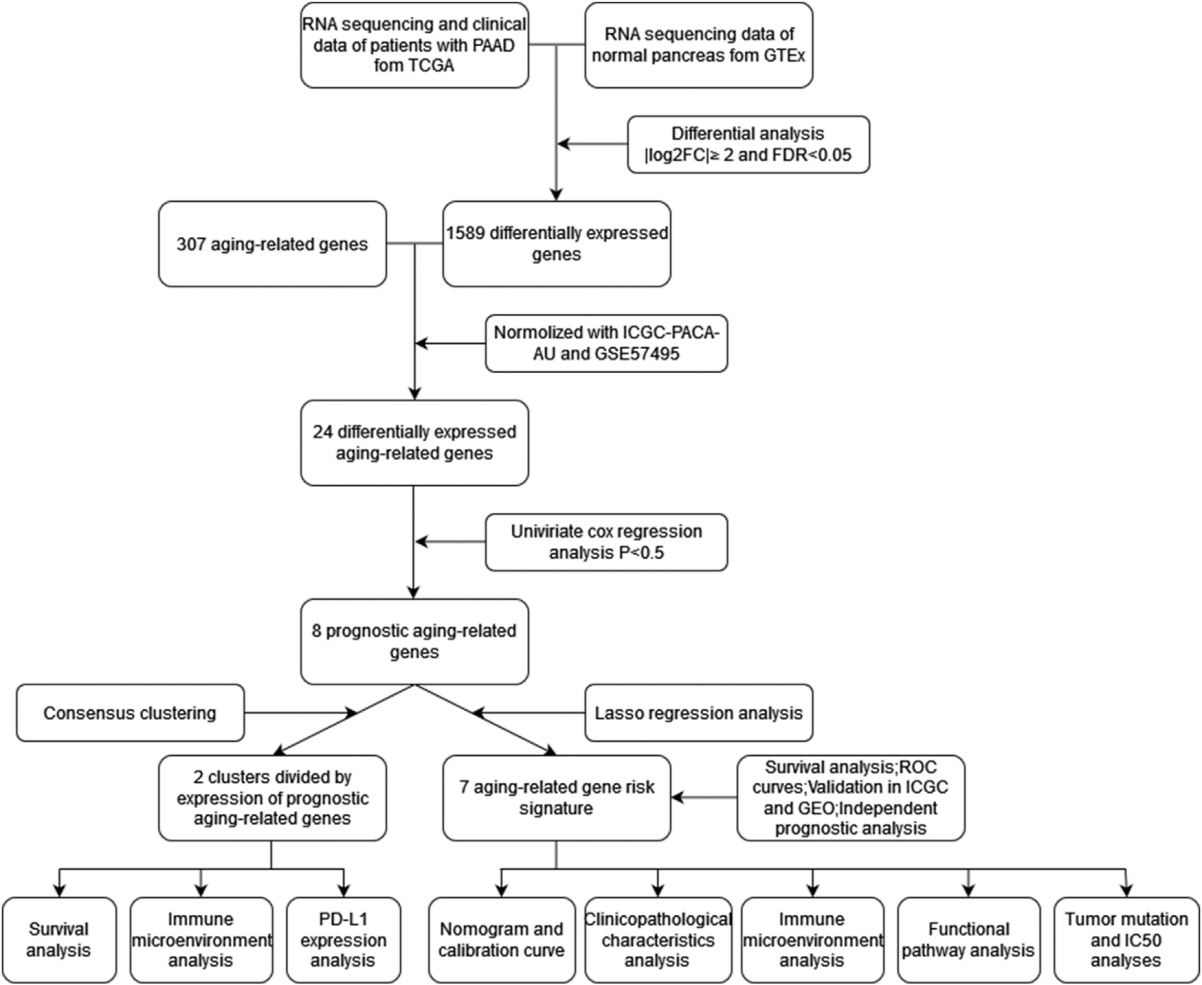
Workflow of the study.

**FIGURE 2:**
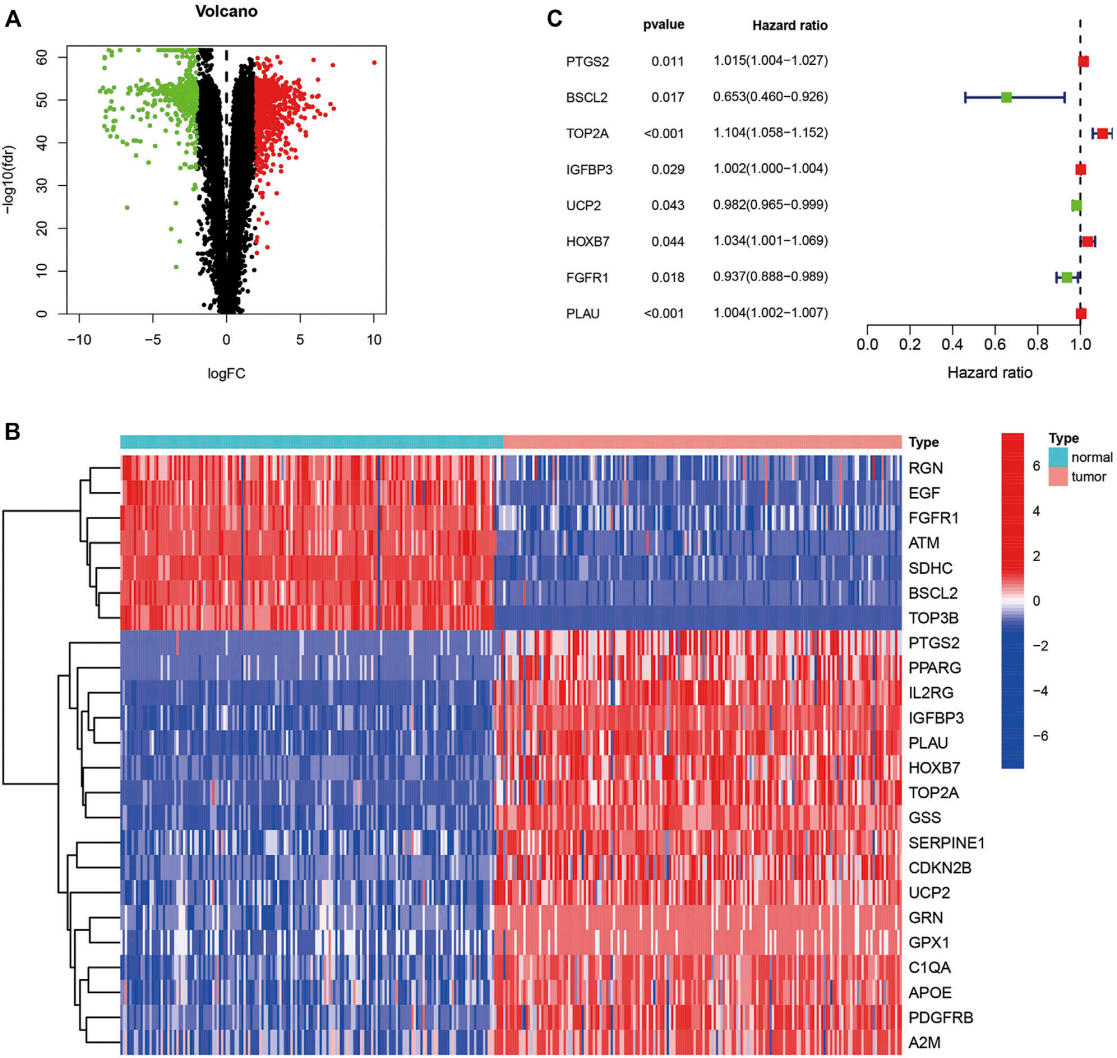
Identification of ARGs with predictive ability. **(A)** Volcano map of differentially expressed genes in TCGA and GTEx. **(B)** Heatmap of differentially expressed ARGs. **(C)** Forest plot presenting the hazard ratios of the prognostic ARGs. ARGs, aging-related genes; TCGA, the cancer genome atlas; GTEx, genotype-tissue expression.

The workflow was generalized in Figure 1. A total of 1,589 DEGs between normal and tumor samples were selected based on both TCGA and GTEx datasets and illustrated in the volcano map (Figure 2A). Furthermore, we acquired 24 ARGs (Supplementary Table S2) from these DEGs, and a heatmap exhibited their differential expression of them (Figure 2B). We implemented a univariate Cox regression analysis and eight ARGs with prognostic values were obtained (Figure 2C, *p* < 0.05). *PTGS2*, *TOP2A*, *IGFBP3*, *HOXB7*, and *PLAU* were dangerous, while *BSCL2*, *UCP2*, and *FGFR1* were protective.

### Survival and immune microenvironment of molecular subtype

**FIGURE 3:**
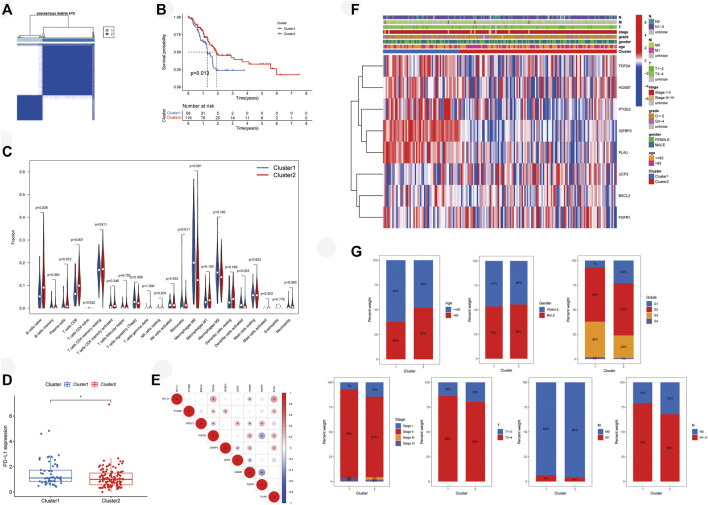
Molecular subtypes of PAAD patients mediated by ARGs. **(A)** Concordance matrix of subtypes. **(B)** Survival analysis demonstrates that cluster 1 has poor survival. **(C)** Vioplot visualizes immune microenvironments of two clusters. **(D)** PD-L1 expressed higher in cluster 1. **(E)** Correlation of PD-L1 with ARGs. **(F)** Heatmap depicts the expressions of prognostic aging-related genes and clinical features of two clusters. **(G)** Comparison of clinical features in two clusters. PAAD, pancreatic adenocarcinoma; ARGs, aging-related genes; PD-L1, programmed cell death-ligand 1; *p < 0.05.

Patients with PAAD from the TCGA cohort were classified into two molecular clusters by consensus clustering with the standard of highest clustering stability (Figure 3A, *k* = 2). OS was lower in cluster 1 (*n* = 58) compared with cluster 2 (*n* = 119) (Figure 3B, *p* < 0.05). Moreover, we evaluated the immune infiltration of each cluster (Figure 3C). Naive B cells (*p* < 0.01), plasma cells (*p* < 0.05), CD8 T-cells (*p* = 0.001), and monocytes (*p* < 0.05) were higher infiltrated in cluster 2, while cluster 1 possessed higher infiltrations of M0 macrophages (*p* < 0.001) and activated dendritic cells (*p* < 0.05). Expression of PD-L1 was higher in cluster 1 (Figure 3D, *p* < 0.05). *TOP2A* (*p* < 0.05) and *PLAU* (*p* < 0.05) were correlated with *PD-L1* positively, while negative correlation was found between *HOXB7* (*p* < 0.05) with *PD-L1* (Figure 3E). In addition, a heatmap depicted the expressions of prognostic aging-related genes (Supplementary Table S3) and clinical features in two clusters (Figure 3F). We furtherly compared the clinical features between two molecular subtypes and found that cluster 1 possessed a higher grade as well as advanced stage and TNM stage of the tumor (Figure 3G).

### Development and verification of an aging-related gene risk signature

**FIGURE 4:**
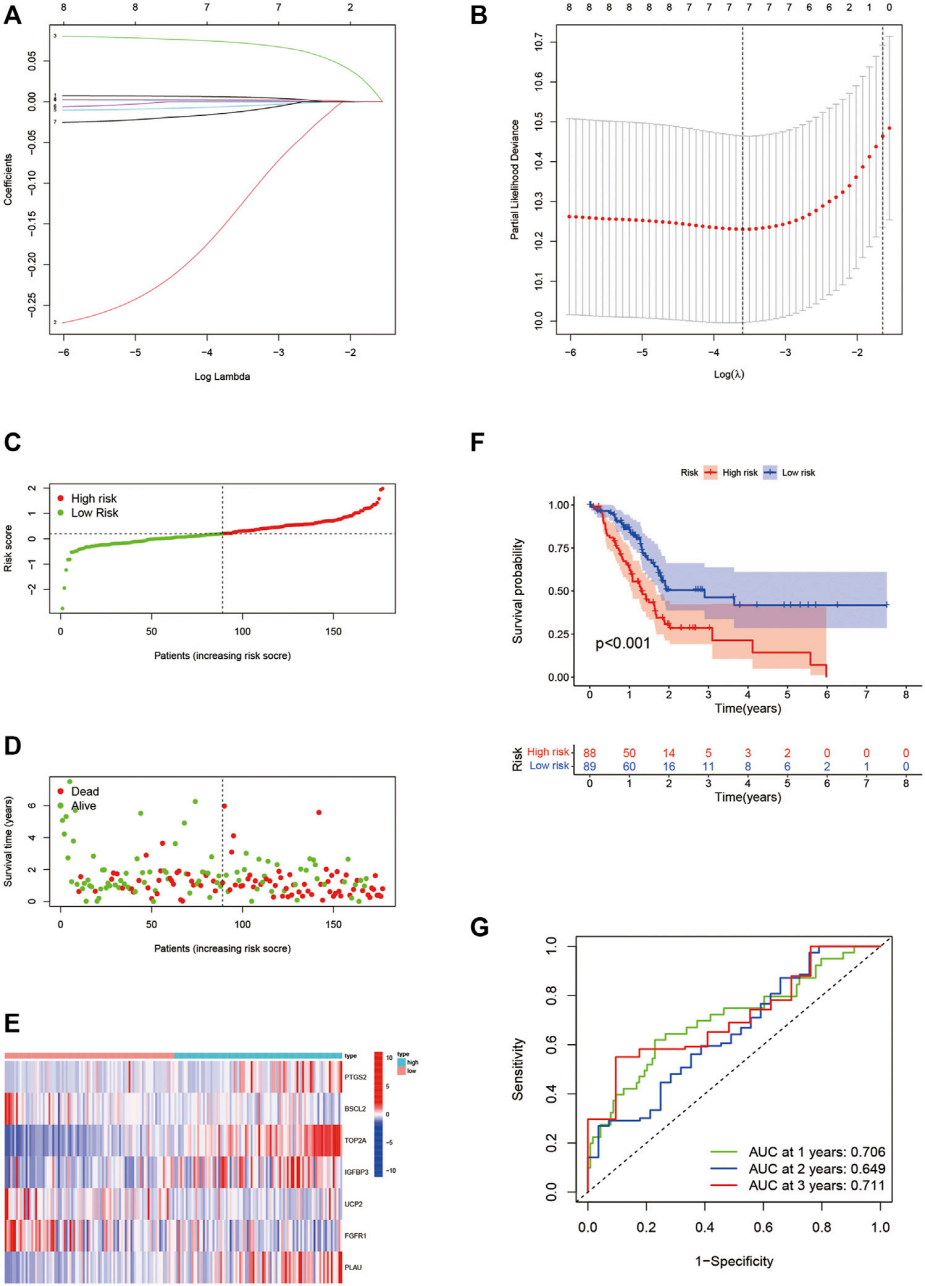
Construction of the seven-ARG risk signature. **(A,B)** LASSO regression analyses for the identification of seven ARGs with the highest prognostic value. **(C)** PAAD patients from the TCGA cohort are sorted by the median risk score. **(D)** Survival conditions of PAAD patients. **(E)** Heatmap of seven ARGs involved in risk signature. **(F)** Kaplan-Meier survival curve displays that the low-risk group has a better survival outcome. **(G)** Time-dependent ROC curves verify the predictive precision of the risk signature. ARGs, aging-related genes; LASSO, Least absolute shrinkage, and selection operator; PAAD, pancreatic adenocarcinoma; TCGA, the cancer genome atlas; ROC, receiver operating characteristic.

**FIGURE 5:**
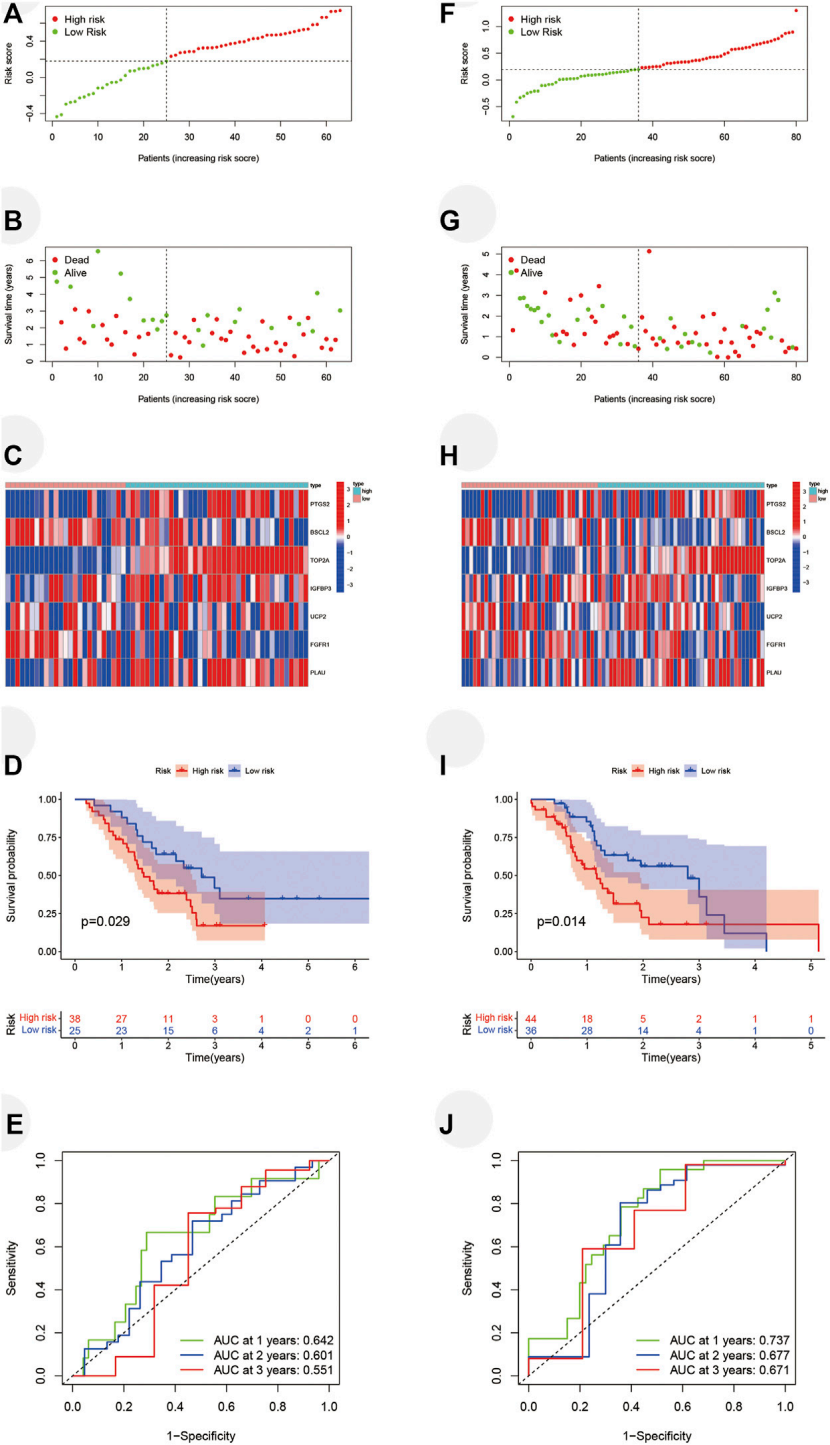
Validation of the seven-ARG risk signature. **(A)** Grouping of PAAD patients from GSE57495 based on the same median risk score. **(B)** Survival status of PAAD patients. **(C)** Heatmap of prognostic ARGs in GSE57495. **(D)** PAAD patients from the high-risk group have terrible survival conditions. **(E)** Time-dependent ROC curves based on GSE57495. **(F–J)** Same validation in ICGC-PCAC-AU. ARG, aging-related gene; PAAD, pancreatic adenocarcinoma; ROC, receiver operating characteristic; ICGC, international cancer genome consortium.

Eight prognostic ARGs were incorporated into the LASSO regression analysis based on the TCGA database (Figures 4A,B). Finally, seven ARGs were identified for the construction of the risk signature (Supplementary Table S4). The risk score was calculated by the sum of each ARG expression multiply each ARG coefficient, and PAAD patients from the TCGA cohort were classified into two risk subgroups by median risk score (Figure 4C). The scatter plot showed the survival rates of patients according to risk scores (Figure 4D), and the heatmap further illustrated the different expressions of seven ARGs between two groups (Figure 4E). High-risk group possessed a poor OS (Figure 4F, *p* < 0.001). Time-dependent ROC curves verified the precision of the risk signature at 1, 2, and 3 years (Figure 4G). All of the aforementioned results were validated in GSE57495 (Figures 5A–E) and ICGC-PACA-AU (Figures 5F–J). Taken together, our ARG risk signature could precisely predict PAAD patients’ survival outcomes.

### Independence analysis of aging-related gene risk signature and establishment of a nomogram

**FIGURE 6:**
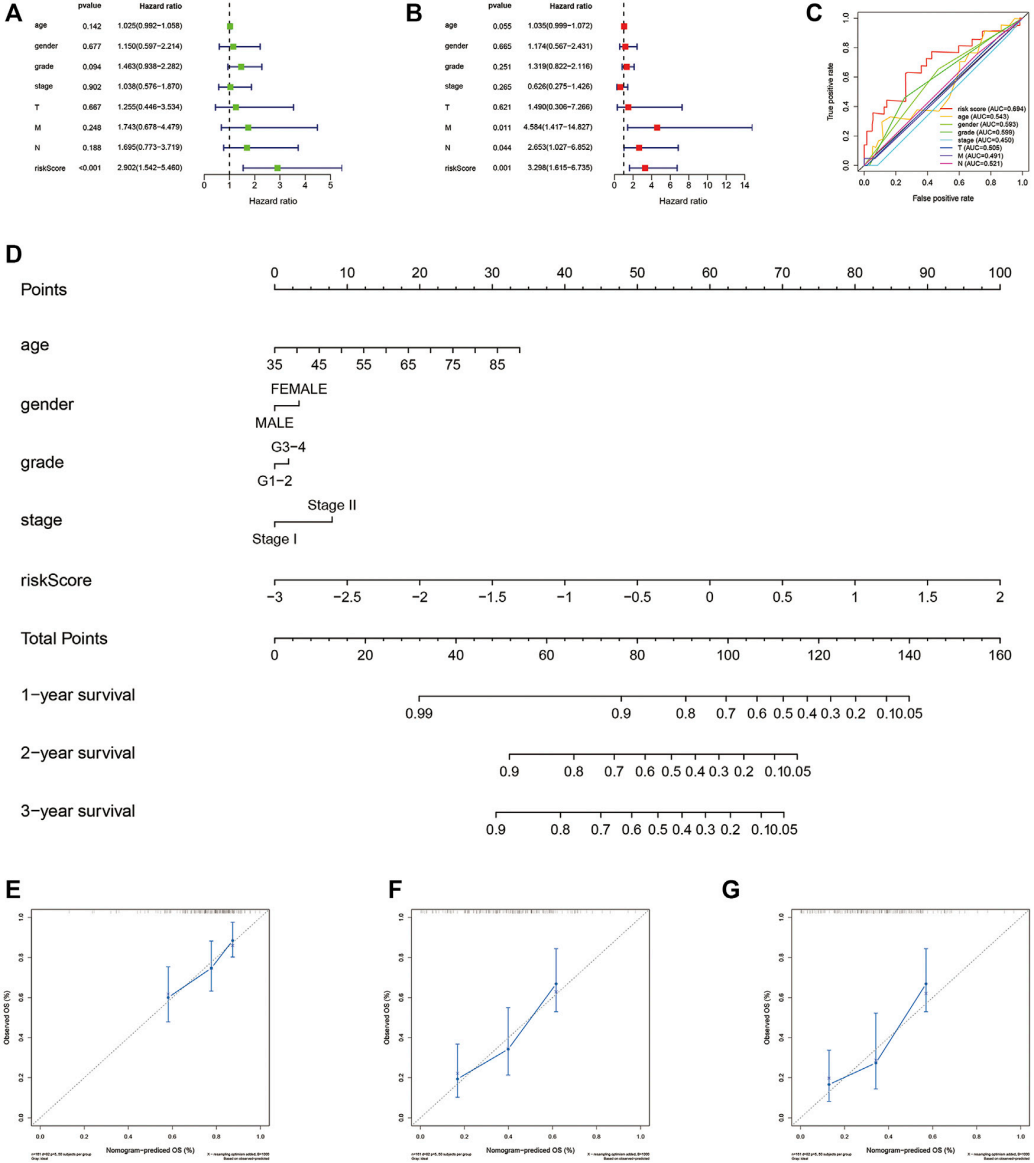
Independence and clinical use of the risk signature. **(A)** Univariate Cox regression analysis based on TCGA database. **(B)**Multivariate Cox regression analysis based on TCGA database. **(C)** Multiple ROC curves depict the predictive precision of risk score and clinicopathological features. **(D)** Nomogram combing risk score and clinical factors. **(E–G)** Calibration curves at 1, 2, and 3 years. TCGA, the cancer genome atlas; ROC, receiver operating characteristic.

We explored whether our risk signature had independently predictive value for PAAD patients from the TCGA cohort. Univariate Cox regression analysis (Figure 6A, Hazard ratio (HR): 2.902, 95% Confidence interval (CI): 1.542–5.406, *p* < 0.001) and multivariate Cox regression analysis (Figure 6B, HR: 3.298, 95% CI: 1.615–6.735, *p* = 0.001) proved it together. Multiple ROC curves verified its predictive stability compared with other clinical factors (Figure 6C). For clinical use, we constructed a nomogram combining risk scores with age, gender, grade, and stage (Figure 6D). Patients at stage III-IV (*n* = 7 of 177) were excluded due to the heterogeneity they brought to the results. Calibration curves were drawn and the precision of our nomogram was well confirmed at 1-, 2-, and 3 years (Figures 6E–G).

### Correlation of risk score with molecular subtype, ImmuneScore, and clinicopathological characteristics

**FIGURE 7:**
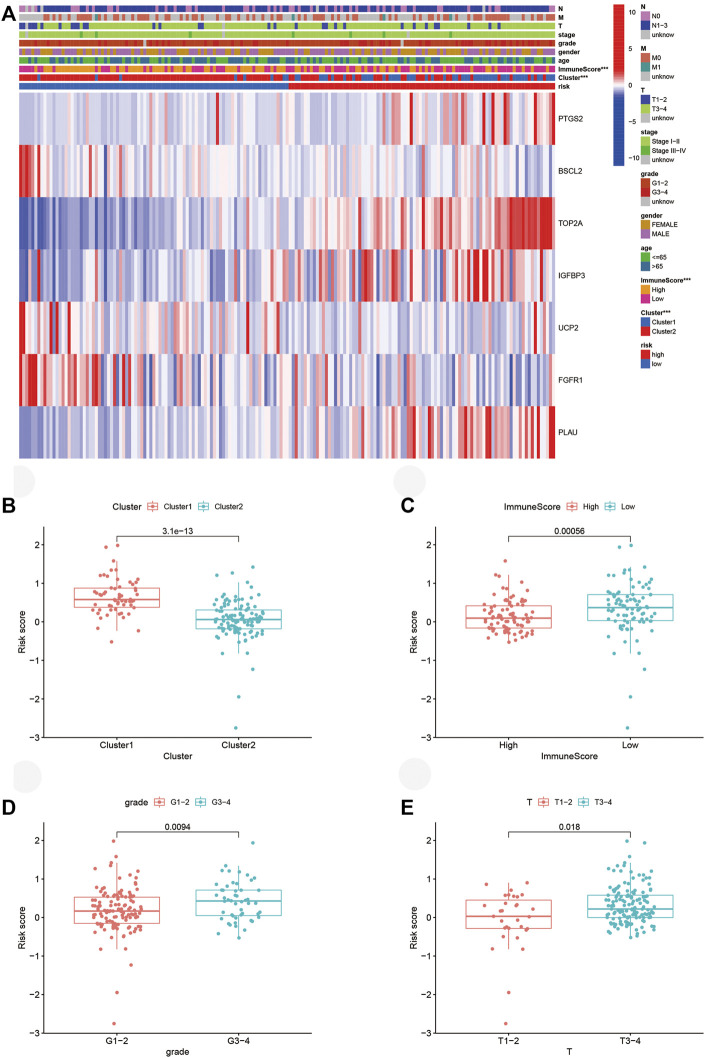
Connections between risk score, molecular subtypes, ImmuneScore, and clinicopathological characteristics. **(A)** Heatmap of ARGs expressed in different subgroups. **(B–E)** Risk score in different clusters, ImmuneScore, grade, and T stage. ARGs, aging-related genes; ***p < 0.001.

The heatmap visualized the associations between ARG expression, risk score, cluster subtype, ImmuneScore, and clinical factor based on the TCGA cohort (Figure 7A). Significant correlations were found between risk score and ImmuneScore as well as the cluster. PAAD patients with high-risk scores took a huge proportion in cluster 1 and possessed lower ImmuneScore. For validation, we explored PAAD patients’ risk scores in different groups. Consistent with aforementioned results, patients from cluster 1 (Figure 7B, *p* < 0.001) and low ImmuneScore subgroup (Figure 7C, *p* < 0.001) retained high risk scores. Interestingly, patients in grades 3-4 (Figure 7D, *p* < 0.01) or Tumor (T) stage 3-4 (Figure 7E, *p* < 0.05) also possessed high-risk scores, which hadn’t been illustrated in the heatmap.

### Immune microenvironment affected by aging-related genes

**FIGURE 8:**
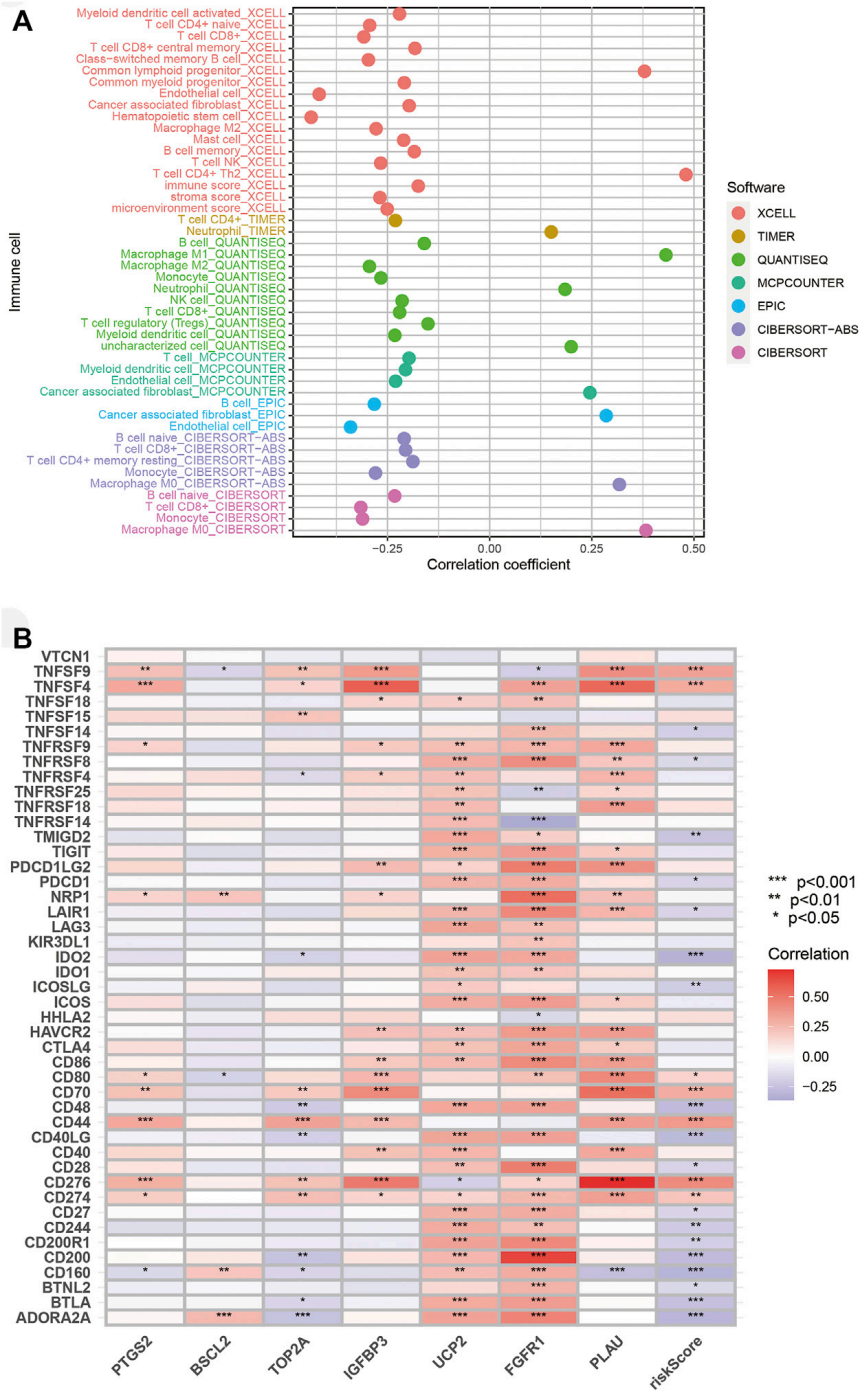
Association of risk score with immune microenvironment. **(A)** Association of immune infiltration with risk score. **(B)** Correlation of immune checkpoint genes expression with risk score. *p < 0.05, **p < 0.01, ***p < 0.001.

Since PAAD patients from the high-risk group achieved lower ImmuneScore, we analyzed their immune microenvironment in detail based on the TCGA cohort. As confirmed by different tests, the risk score was positively associated with M0 macrophage, M1 macrophage, neutrophil, and cancer-associated fibroblast (CAF), while the correlations of NK cell, CD8 T-cell, CD4 T-cell, and B cell with risk score were negative (Figure 8A). Immune score, stroma score, and microenvironment score were also negatively relevant to risk score, demonstrating the terrible immune microenvironment in the high-risk group. Furthermore, we conducted analyses of immune checkpoint gene expression. Expressions of *TNFSF4*, *TNFSF9*, *CD80*, *CD70*, *CD44*, *CD274* (*PD-L1*), and *CD276* were positively correlated with a risk score, while other significant correlations were negative (Figure 8B). These significant correlations could provide options for ICIs in clinical treatment for PAAD.

### Analyses of pathways, tumor mutation, and half inhibitory centration

**FIGURE 9:**
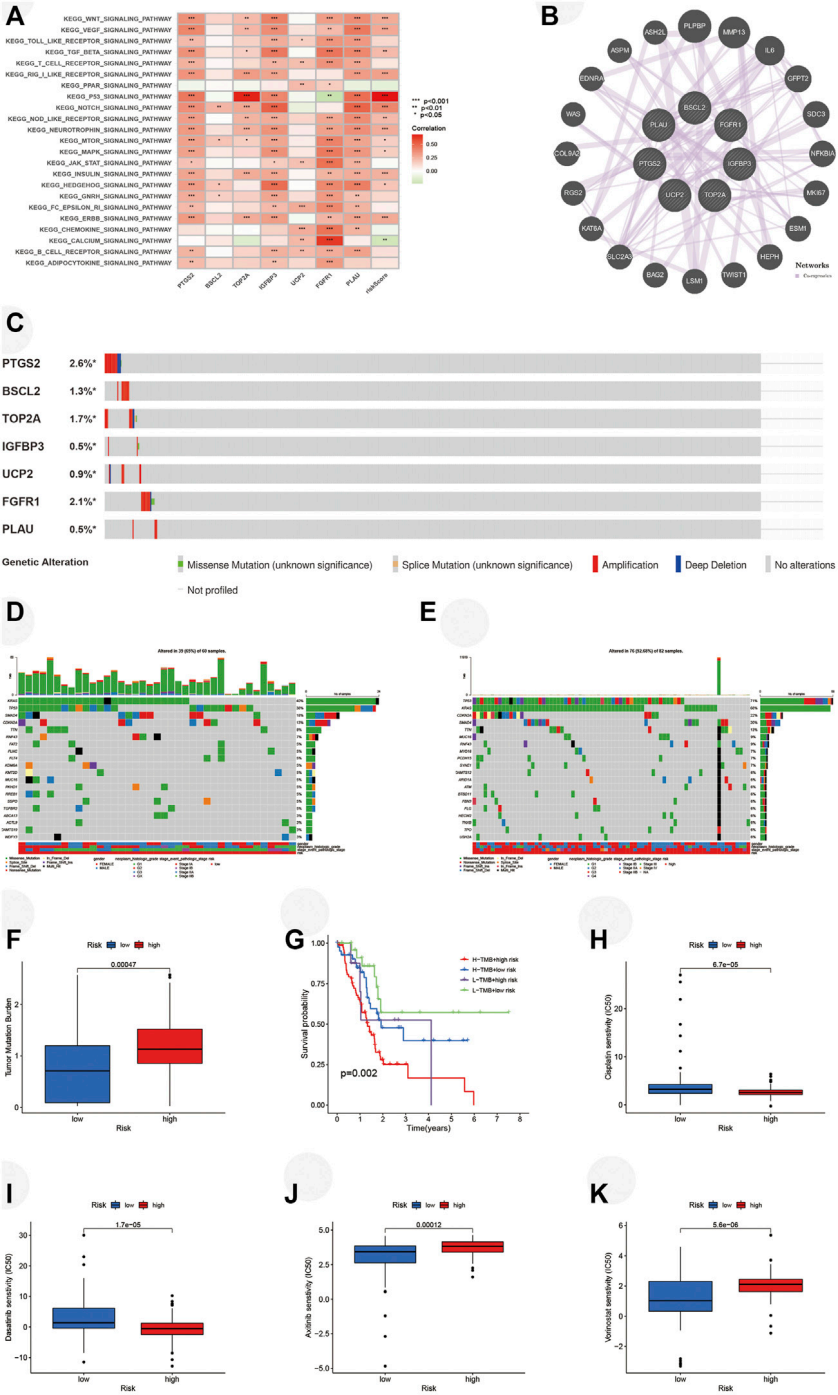
Analyses of pathways, tumor mutation, and IC50 in two risk groups. **(A)** GSVA performed in risk signature based on TCGA. **(B)** Twenty functionally similar genes co-expressing with key ARGs. **(C)** Mutation of key ARGs in PAAD patients. **(D)** Mutation of top 20 genes in the low-risk group. **(E)** Mutation of top 20 genes in the high-risk group. **(F)** TMBs of PAAD patients in two risk groups. **(G)** Survival analysis illustrates that PAAD patients with higher TMB have worse OS in each risk group. Chemosensitivities of **(H)** Cisplatin, **(I)** Dasatinib, **(J)** Axitinib, and **(K)** Vorinostat in two risk groups. IC50, half inhibitory centration; GSVA, gene set variation analysis; TCGA, the cancer genome atlas; ARGs, aging-related genes; TMBs, tumor mutation burdens; PAAD, pancreatic adenocarcinoma; OS, overall survival; *p < 0.05, **p < 0.01, ***p < 0.001.

For the exploration of the potential pathways related to the risk score, we performed GSVA. Several pathways related to the development of PAAD such as *WNT*, *VEGF*, *MTOR*, and *MAPK* pathways were positively associated with a risk score, and the *p53* pathway was most closely correlated with a risk score (Figure 9A). A total of 20 functionally similar genes co-expressing with key ARGs were found by GeneMANIA (Figure 9B). Mutations of seven key ARGs were investigated in 849 PAAD patients and found in 62 (7%) of them (Figure 9C). *PTGS2* mutation accounted for the largest and amplification was the main form of genetic alteration. We then analyzed the mutations of the top 20 genes in two risk groups. The oncoplots illustrated that the high-risk group retained higher genetic mutation (Figures 9D,E). Furthermore, we identified that the high-risk group bore a higher TMB (Figure 9F). Survival analysis demonstrated that patients with lower TMBs got better OS compared with those with higher TMBs in either risk group (Figure 9G). Finally, we explored the ability of our risk signature to predict the chemosensitivities of several drugs for PAAD. On the basis of the prediction made by the pRRophetic algorithm, patients from the high-risk group may be more sensitive to cisplatin (Figure 9H, *p* < 0.001) and dasatinib (Figure 9I, *p* < 0.001), while axitinib (Figure 9J, *p* < 0.001) and vorinostat (Figure 9K, *p* < 0.001) may be better options for the low-risk group.

### Protein and mRNA expression level of aging-related genes and survival analysis

**FIGURE 10:**
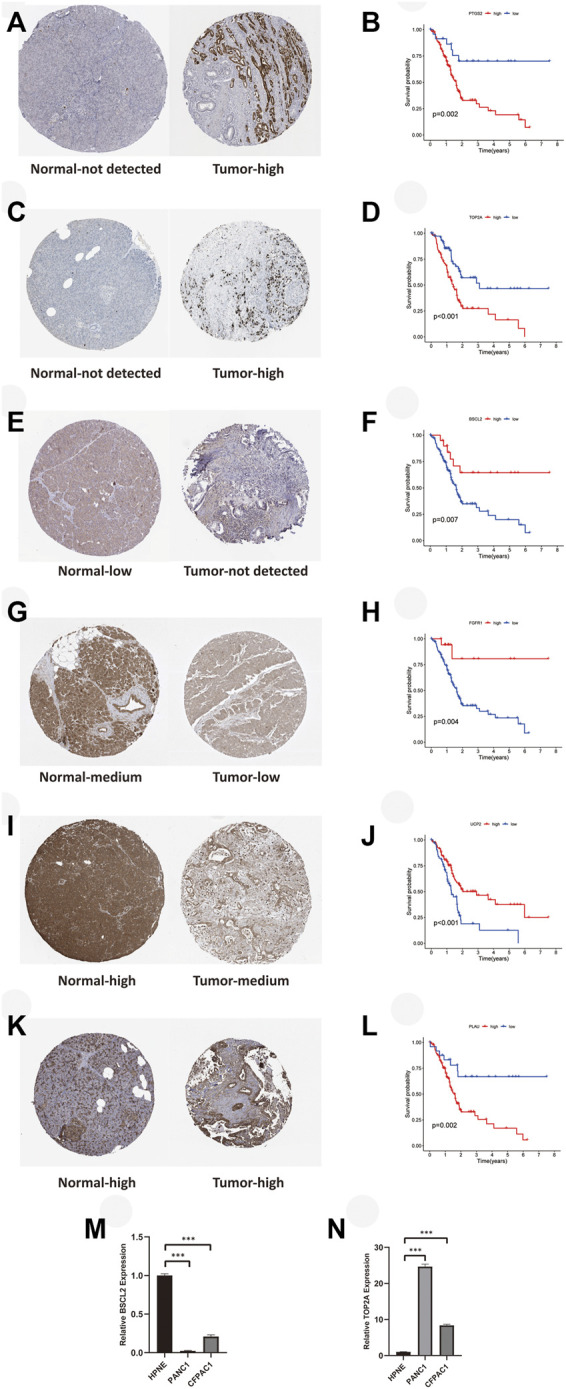
Immunohistochemical and RT-qPCR results of key ARGs expressions with their influences on OS. **(A–B)** PTGS2. **(C–D)** TOP2A. **(E–F)** BSCL2. **(G–H)** FGFR1. **(I–J)** UCP2. **(K–L)** PLAU. **(M)** BSCL2 is expressed higher in the normal cell line. **(N)** TOP2A is expressed higher in tumor cell lines. RT-qPCR, real time quantitative polymerase chain reaction; ARGs, aging-related genes; OS, overall survival; *p < 0.05, **p < 0.01, ***p < 0.001.

For validation of ARG expression in normal and tumor tissues, we acquired immunohistochemical results from HPA. Except for IGFBP3 which hadn’t been found in HPA, as we predicted, protein expressions of *PTGS2* (Figures 10A,B, *p* < 0.01) and *TOP2A* (Figures 10C,D, *p* < 0.001) were higher in pancreatic tumor tissue, which was consistent with their correlation with poor survival of PAAD patients. *BSCL2* (Figures 10E,F, *p* < 0.01), *FGFR1* (Figures 10G,H, *p* < 0.01), and *UCP2* (Figures 10I,J, *p* < 0.001) were expressed higher in normal pancreatic tissue, and their higher expressions were relevant to better survival outcomes. It was worth noting that *UCP2* was expressed higher in tumor samples based on TCGA and GTEx, which was contrary to its immunohistochemical result. The exact result should be verified in a large cohort further. *PLAU* (Figure 10K,L, *p* < 0.01) was expressed higher both in normal and tumor tissues, but its association with worse OS was consistent with what we found before. Finally, we verified the expression of the key ARGs in normal and tumor pancreatic cell lines. Compared with HPNE, *BSCL2* was expressed lower in PANC1 and CFPAC1 (Figure 10M, *p* < 0.001), while *TOP2A* was expressed higher (Figure 10N, *p* < 0.001).

## Discussion

Aging, as an inevitable decline of physiological function, has shown tight correlations with the incidence and death rate of cancer. As the main risk factor of cancer, aging resulted in the generation of inflammatory mediators, which were considered a crucial part of the tumor microenvironment and a trigger of tumor (Bottazzi et al., 2018). The decline of the immune system in elder individuals also weakens their resistance to tumorigenesis (Franceschi and Campisi, 2014). In terms of immune microenvironment, a previous study has proved that M2 tumor-associated macrophages were infiltrated in the microenvironment of aged mice, which led to immunosuppression and promotion of tumors (Jackaman et al., 2013). Moreover, increased expression of *PD-L1*, as well as immunosenescence of effector immune cells, has also been found in aged mice (Golomb et al., 2015). However, cellular senescence also plays an antitumor role in malignancy, which might be induced as a therapeutic (Collado et al., 2005; te Poele et al., 2002). The complex correlation of aging with tumors prompts us to explore the relationship between ARGs and cancer. Yue et al. (2021) have identified an ARG signature to predict immune infiltration as well as the prognosis of colorectal cancer. Nevertheless, the survival outcome and immune microenvironment characterization influenced by ARGs have not been completely elucidated in PAAD.

In this study, combing the TCGA dataset with the GTEx dataset, we first obtained eight differentially expressed ARGs with prognostic values and then identified two molecular subtypes mediated by them. There existed distinct differences in OS and immune infiltrations between the two clusters. Compared to patients in cluster 2, patients in cluster 1 had less effector immune cell infiltrations and worse OS. Moreover, we found that cluster 1 had a higher grade and advanced stage of the tumor, which may explain its poor survival.

We also constructed and validated a risk signature to investigate ARGs’ predictive ability of prognosis and immune microenvironments of PAAD patients from the TCGA cohort. LASSO regression analysis was used to screen robust prognostic biomarkers to establish an effectively prognostic ARG risk signature. The most important seven key ARGs (*PTGS2*, *BSCL2*, *TOP2A*, *IGFBP3*, *UCP2*, *FGFR1*, *PLAU*) were finally identified. These key aging-related genes play different roles in aging. Senescence surveillance, immune-mediated destruction of senescence cells, is partly dependent on *PTGS2* and its product *PGE2* (Gonçalves et al., 2021). Seipin is a protein encoded by *BSCL2*. Seipin plays a crucial role in the synthesis of lipid droplets, which interact with peroxisomes, key organelles in aging, and central degenerative disease (Sánchez-Iglesias et al., 2021). *TOP2A* encodes DNA topoisomerases to maintain the stability of DNA replication, ensuring normal cell proliferation (Wang, 2002). *IGFBP3* and *UCP2* are both related to muscle phenotypes associated with sarcopenia, an age-related loss of muscle function (Pratt et al., 2019). Aging suppressor αKlotho binds to *FGF23* through *FGFR1*, and the ternary complexes are involved in the regulation of phosphate and vitamin D homeostasis (Chen et al., 2018). *PLAU*, closely connected with lots of physiologic and pathologic processes, such as inflammatory, *p53* signal pathway, invasion, cell proliferation, and apoptosis, is directly associated with aging and age-related diseases such as Alzheimer’s disease and complications of diabetes (Cardoso et al., 2018). Senescent cells expressed and secreted *PLAU* to mediate cell proliferation and apoptosis (Hildenbrand et al., 2008).

Some biomarkers involved in our gene signature have been studied in pancreatic cancer. A previous study proved that *TOP2A* accelerated the development of pancreatic cancer through the activation of the β-catenin pathway (Pei et al., 2018). Similarly, *DGCR5* was proved that can promote pancreatic cancer by targeting *miR-3163*/*TOP2A* (Liu et al., 2021). Moreover, Mody et al. (2019) suggested increasing expression of *TOP2A* was found in *PD-L1* positive tumors. In our study, correlation evaluation also demonstrated that *TOP2A* expression was positively relevant to *PD-L1*, implying that *TOP2A* could be a dangerous biomarker and a potential target of treatment for PAAD.

PAAD patients with higher risk scores had worse OS compared with patients in the low-risk group. ROC curves verified the prognostic precision of ARG risk signature for 1-, 2-, and 3-year OS. After controlling confounding factors, our risk score was proved to independently predict PAAD patients’ OS. For clinical application, we established a nomogram combining risk scores with other clinical features, which facilitated the availability of the ARG risk signature. Moreover, the risk score was remarkably relevant to the ImmuneScore, grade, and T stage of PAAD patients as well as their molecular subtypes. Taken together, the ARG risk signature had a reliably predictive value for the prognosis of PAAD patients.

The immune microenvironment is a crucial component of cancer as well as a therapeutic target for PAAD. The immune microenvironment is primarily infiltrated by stromal and immune cells, and evaluations of them are associated with clinical signatures and prognosis in PAAD (Quail and Joyce, 2013; Pitt et al., 2016). In our study, negative correlations were found between risk score and effector immune cells (such as CD4^+^ T-cells, CD8^+^ T-cells, and NK cells) infiltrations. Moreover, a higher risk score was relevant to lower immune, stroma, and estimate scores. These results certified that immune microenvironments of PAAD patients with higher risk scores were worse, which possibly explained their unfavorable OS. CAFs were important components of the immune microenvironment and several promoted while others suppressed the development of PAAD (Geng et al., 2021). We found that CAFs were positively correlated with a risk score. Therefore, it is necessary to classify the subtypes of CAFs infiltrated in the immune microenvironments of PAAD patients, and perform subgroup analyses further to verify their relationships with a risk score. Overall, our ARG risk signature could be used for immune microenvironment stratification of patients with PAAD.

In addition, the risk score was also relevant to the expressions of several immune checkpoint genes positively. Inhibitor of *PD-L1* was recognized as effective antitumor immunotherapy for different kinds of cancer, including pancreatic cancer. Moreover, *CD44* was expressed higher in several solid tumors, such as pancreatic cancer, which was considered an important target for therapeutic (Mattheolabakis et al., 2015). *CD276* also retained high expression in pancreatic cancer and was associated with worse OS (Inamura et al., 2018). Therefore, our research provided potential immunotherapeutic targets for PAAD patients based on their risk score, and targeting aging could serve as a valuable regulation for immunotherapy in PAAD.

Aging is correlated with several biological processes of various tumors, including PAAD (Campisi, 2013; Campisi and d’Adda di Fagagna, 2007). We further explored potential pathways related to risk score positively by GSVA. Several molecules promoted tumorigenesis, progression, and chemoresistance of pancreatic cancer through activation of the *wnt*/β-catenin pathway (Zhou et al., 2020; Chen et al., 2021). Oxymatrine played a role in antiangiogenesis in PAAD by inhibiting the *VEGF* pathway (Chen et al., 2013). Moreover, the *mTOR* pathway was also activated in pancreatic cancer to the promotion of progression and drug resistance (Mann et al., 2016). The tightest link was found between the *p53* pathway and risk score, and inactivation of *p53* was found in almost every tumor (Joerger and Fersht, 2016). Zhang et al. (2018) have proved that blockade of cell proliferation and acceleration of cell senescence could be triggered by *CCNB1* silencing through activation of the *p53* pathway. These correlations between risk scores and pathways could offer several potential therapeutic targets to PAAD patients, while more details should be validated by fundamental experiments. In addition, we found 20 functionally similar genes of key ARGs. Their potential functions and pathways needed to be explored further.

The accumulation of gene mutation leads to tumorigenesis. Thus, we first investigated the mutations of key ARGs in PAAD patients and found the mutations were rare. Moreover, we found significant differences in genetic alterations among the two risk groups. Higher genetic mutations were found in patients from the high-risk group, such as *KRAS*, *TP53*, *SMAD4*, and *CDKN2A*, which were major drivers of PAAD (Jones et al., 2008; Waddell et al., 2015; Makohon-Moore and Iacobuzio-Donahue, 2016). Patients with high-risk scores bore higher TMBs. The correlation of higher TMB with better efficacy of immunotherapy has been proved (Samstein et al., 2019). Thus, patients from the high-risk group may benefit from immunotherapy, which should be confirmed in clinical further. In addition to the poor survival in the high-risk group, higher TMB was also related to worse OS. However, TMB could not be an effectively prognostic biomarker on its own (Addeo et al., 2021). More clinical trials should be implemented to explore the relationship between TMB and OS.

PAAD shows resistance to chemotherapy due to complex factors (Springfeld et al., 2019). Therefore, we explored the potential sensitive drugs for PAAD patients from different risk subgroups. Since patients with different risk scores were predicted to be sensitive to different drugs, our risk signature could offer several therapeutic options for them. The final effect still needed to be verified in clinical trials.

Finally, we used immunohistochemical results from HPA to confirm the expressions of key ARGs. Their expressions in normal and tumor samples were in keeping with their influences on OS. However, *UCP2* showed a contrary tendency in expression compared with that in the TCGA dataset. Moreover, the result of *IGFBP3* was not found in *HPA*, and *PLAU* didn’t express differentially in normal and tumor samples. In the future, we hope to validate their expressions ourselves. The results of RT-qPCR verified the different mRNA levels of key aging-related genes in normal and tumor cells, which was consistent with our previous analyses.

There were some limitations existing in our study. On the one hand, more experiments *in vivo* and vitro were needed for verification of the results we identified by bioinformatic analyses. We hope to conduct functional validation of the prognostic ARGs we found in the future. On the other hand, PAAD samples from TCGA, ICGC, and GEO were deficient, thus we needed more samples from a large cohort to verify our risk signature further.

## Conclusion

We identified two molecular subtypes and established a novel ARG risk signature based on ARGs. According to the risk score generated from our signature, we predicted the prognoses, immune microenvironments, tumor mutations, and drug sensitivity of PAAD patients. Our study may offer a novel understanding of the molecular mechanism in PAAD as well as new targets and options for the clinical treatment of PAAD.

## Data Availability

The datasets presented in this study can be found in online repositories. The names of the repository/repositories and accession number(s) can be found in the article/Supplementary Material.

## Abbreviations

PAAD, pancreatic adenocarcinoma; OS, overall survival; ICIs, immune checkpoint inhibitors; ARGs, aging-related genes; TCGA, the cancer genome atlas; GTEx, genotype-tissue expression; GEO, gene expression omnibus; ICGC, international cancer genome consortium; HAGR, human aging genomic resources; HPA, human protein atlas; DEGs, differentially expressed genes; PD-L1, programmed cell death-ligand 1; LASSO, least absolute shrinkage and selection operator; ROC, receiver operating characteristic; GSVA, gene set variation analysis; TMB, tumor mutation burden; IC50, half inhibitory centration; RT-qPCR, real time quantitative polymerase chain reaction; HR, hazard ratio; CI, confidence interval; CAF, cancer associated fibroblast
